# Epidemiologic Characteristics, Prognostic Factors, and Treatment Outcomes in Primary Central Nervous System Lymphoma: A SEER-Based Study

**DOI:** 10.3389/fonc.2022.817043

**Published:** 2022-02-10

**Authors:** Dongsheng Tang, Yue Chen, Yuye Shi, Hong Tao, Shandong Tao, Quan’e Zhang, Banghe Ding, Zhengmei He, Liang Yu, Chunling Wang

**Affiliations:** ^1^Department of Hematology, The Huaian Clinical College of Xuzhou Medical University, Huai’an, China; ^2^Department of Hematology, The Affiliated Huaian No.1 People’s Hospital of Nanjing Medical University, Huai’an, China

**Keywords:** primary central nervous system lymphoma, SEER, treatment, prognosis, nomogram

## Abstract

**Objective:**

This study was conducted in order to study the clinical characteristics, prognostic factors, and treatment outcomes in patients with primary central nervous system lymphoma (PCNSL).

**Materials and Methods:**

The data of a total of 5,166 PCNSL patients diagnosed between 2000 and 2018 from the Surveillance, Epidemiology, and End Results (SEER) database were obtained.

**Results:**

The mean age was 63.1 ± 14.9 years, with a male to female ratio of 1.1:1.0. The most common histologic subtype was diffuse large B-cell lymphoma (DLBCL) (84.6%). The 1-, 3-, and 5-year overall survival (OS) rates were 50.1%, 36.0%, and 27.2%, respectively, and the corresponding disease-specific survival (DSS) rates were 54.4%, 41.3%, and 33.5%, respectively. Multivariate analysis with Cox regression showed that race, sex, age, marital status, surgical resection, and chemotherapy were independent prognostic factors for OS and DSS, but radiotherapy was only for OS. Nomograms specially for DLBCL were established to predict the possibility of OS and DSS. The concordance index (C-index) values of OS and DSS were 0.704 (95% CI 0.687–0.721) and 0.698 (95% CI 0.679–0.717), suggesting the high discrimination ability of the nomograms.

**Conclusion:**

Surgical resection and/or chemotherapy was favorably associated with better OS and DSS. However, radiotherapy was not beneficial for OS and DSS in the long term. A new predictive nomogram and a web-based survival rate calculator we developed showed favorable applicability and accuracy to predict the long-term OS for DLBCL patients specifically.

## Introduction

Primary central nervous system lymphoma (PCNSL) is an uncommon and highly invasive tumor that involves the leptomeninges, brain, eyes, or spinal cord without evidence of systemic disease (1). PCNSL accounts for 1%–2% of non-Hodgkin lymphoma (NHL) and most of the cases (over 90%) are diffuse large B-cell lymphoma (DLBCL) (2, 3). Immunocompromised individuals, such as HIV-infected or immunosuppressive patients, are deemed to have a higher risk of developing PCNSL (4, 5). PCNSL was historically associated with poor prognosis, with an overall survival (OS) of 1.5 months if the patient did not receive proper treatment. High-dose methotrexate (HD-MTX) systemic chemotherapy is deemed as the standard first-line treatment; however, few patients can achieve long-term survival: the median progression‐free survival (PFS) and OS were only 24.0 and 36.9 months, respectively (6, 7), and the 5-year OS in the period 1992–1994 was increased only from 19.1% to 30.1% in the period 2004–2006 (4). Surgical resection was generally discouraged before 2010, but the conventional view has been challenged with the development of surgical techniques. Due to the high risk of neurotoxicity and the lack of sustainable response, whole-brain radiation (WBRT) is no longer considered as the first-line treatment. More research has focused on whether different radiotherapy regimens (including reduced dose and partial‐brain radiotherapy) in combination with chemotherapy can bring benefits. However, the conclusions are inconsistent. In recent years, novel agents including immune checkpoint inhibitors, immunomodulatory drugs (IMiDs), Bruton tyrosine kinase (BTK) inhibitor, PI3K/AKT/mTOR inhibitors, and chimeric antigen receptor T cell (CAR-T cell) therapy have been applied in several clinical trials, which exhibit promising clinic outcomes. Even with an impressive clinical response (8–11), more randomized clinical trials are still needed to verify and identify the optimal therapy for PCNSL patients.

Two prognostic classification systems of PCNSL are widely used currently. The IELSG identified that age (>60 years), elevated lactate dehydrogenase (LDH) serum level, performance status (PS) (≥2), high CSF protein concentration, and extensive deep structure involved in the brain were independent predictors of negative prognosis (12). In another prognostic model, the MSKCC prognostic score described three risk groups based on age and Karnofsky performance status (KPS) (13). However, treatment information was not included in these prognostic systems, and it was difficult to perform the treatment options based on these prognostic systems. Therefore, to develop a new, easily available prognostic system that includes treatment information is needed.

Due to the low incidence of PCNSL, large-scale clinical trials and prospective data are limited for us to investigate. The Surveillance, Epidemiology, and End Results (SEER) contains a wealth of relevant information on different types of cancer patients based on the United States population which provides excellent resources for us to study. Therefore, a large population-based analysis was conducted to describe the clinical characteristics, prognostic factors, and treatment outcomes of PCNSL using the SEER database, and based on the independent prognostic factors of PCNSL, a nomogram was established to predict the prognosis of PCNSL patients.

## Materials and Methods

Study data were obtained using the SEER*Stat software (version 8.3.9). Through the “Incidence-SEER Research Plus Data, 18 Registries, Nov 2020 Sub (2000–2018),” patients diagnosed with PCNSL between 2000 and 2018 were identified. The International Classification of Diseases for Oncology (ICD-O-3) histologic codes (9590–9595, 9650–9699, 9702–9729) were used for lymphoma and primary sites limited to the central nervous system were identified by site-specific codes (C71.0–C71.9). Unknown age at diagnosis, race, sex, marriage status, incomplete follow-up data, and secondary to other tumors were excluded. The primary endpoint of this study was OS and DSS.

### Statistical Analyses

The OS and DSS were estimated with the Kaplan–Meier method using the log-rank test, and the Cox regression model was used for univariate and multivariate survival analyses. Nomograms were constructed to predict the 1-, 3-, and 5-year OS and DSS specifically for DLBCL according to the results of multivariable Cox regression analysis. To evaluate the accuracy of the nomograms, Harrell’s concordance index (C-index) was calculated to quantify the discrimination performance, and the calibration curve was plotted to identify whether the predicted survival was consistent with the actual survival. The data were analyzed using R software (R version 4.0.4). Statistical significance was set at *P <*0.05 (two-sided).

## Results

### Epidemiologic Characteristics of PCNSL Patients

Totally, 5,166 PCNSL patients were included in the study. The mean age at diagnosis was 63.1 ± 14.9, ranging from 3 to 97 years. The population was comprised of 2,679 (51.9%) males and 2,487 (48.1%) females, and the highest incidence of age group was 70–79 years old. The characteristics of PCNSL patients are summarized in **Table 1**.

**Table 1:** Patient and tumor characteristics of primary central nervous system lymphoma diagnosed in SEER 18 registries, 2000–2018.

Characteristic	No. of patients	Percentage (%)
Total	5,166	100
Age at diagnosis, years		
Mean ± SD	63.1 ± 14.9	
Median (range)	65.0 (3.0–97.0)	
Sex		
Male	2,679	51.9
Female	2,487	48.1
Race		
White	4,182	81.0
Black	364	7.0
Others^a^	620	12.0
Years of diagnosis		
2000–2008	2,115	40.9
2009–2018	3,051	59.1
Age		
<50	916	17.7
50–59	884	17.1
60–69	1,372	26.6
70–79	1,415	27.4
≥80	579	11.2
Marital status		
Married	3,045	58.9
Single^b^	2,121	41.1
Lineage		
Aggressive B-cell NHL^c^	4,429	85.7
Indolent B-cell NHL^d^	166	3.2
T-cell NHL	85	1.6
NHL-NOS	474	9.2
Others	12	0.2
Surgery		
No/unknown	3,040	58.8
Performed	2,126	41.2
Radiation		
No/unknown	3,608	69.8
Performed	1,558	30.2
Chemotherapy		
No/unknown	1,652	32.0
Performed	3,514	68.0

NHL, non-Hodgkin lymphoma; NOS, not otherwise specified.

a American Indian/Alaskan Native or Asian/Pacific Islander.

b Included divorced/separated/widowed patients.

c Included diffuse large B-cell lymphoma, Burkitt’s lymphoma, mantle cell lymphoma, and intravascular large B-cell lymphoma.

d Included follicular lymphoma, chronic lymphocytic leukemia/small lymphocytic lymphoma, lymphoplasmacytic lymphoma, and mucosa-associated lymphoid tissue lymphoma.

Regarding clinical aggressiveness and cell of origin, 4,429 (85.7%) patients had aggressive B-cell NHLs, 166 (3.2%) had indolent B-cell NHLs, 474 (9.2%) had NHL-NOSs, and 85 (1.6%) had T-cell NHLs. As for the histological classification of PCNSL, the most common subtype was DLBCL (84.6%), followed by NHL not otherwise specified (NHL-NOS) (9.2%), follicular lymphoma (FL) (1.3%), peripheral T-cell lymphoma (PTCL) (1.2%), mucosal-associated lymphoid tissue (MALT) (1.2%), Burkitt’s lymphoma (BL) (0.7%), lymphoplasmacytic lymphoma (LPL) (0.4%), anaplastic large cell lymphoma (ALCL) (0.4%), and chronic lymphocytic leukemia/small lymphocytic lymphoma (CLL/SLL) (0.4%). Except for ALCL (median age 39.0), the median age for all the other subtypes was over 60 years old. The epidemiologic characteristics and survival outcomes are summarized according to histological subtype in **Table 2**.

**Table 2:** Patient characteristics according to histological subtypes.

Histology subtype	*n* (%)	Median age	Male (%)	Survival			
				Median OS, m	3-Year OS	Median DSS, m	3-Year DSS
All patients	5,166						
DLBCL	4,373 (84.6)	66.0	51.8	12	35.3	17	40.5
NHL-NOS	474 (9.2)	65.0	54.0	8	32.0	11	37.3
FL	65 (1.3)	67.0	41.5	58	53.8	77	57.4
PTCL	63 (1.2)	60.0	57.1	15	37.9	26	47.3
MALT	60 (1.2)	60.0	31.7	/	78.3	/	86.0
BL	34 (0.7)	60.0	64.7	19	38.3	19	42.3
LPL	22 (0.4)	63.0	45.5	/	50.9	/	50.9
ALCL	21 (0.4)	39.0	71.4	13	34.4	22	37.2
CLL/SLL	19 (0.4)	66.0	47.4	29	42.1	47	54.0
Others	35 (0.7)	62.0	54.3	/	/	/	/

n, number of cases; m, months; OS, overall survival; DSS, disease-specific survival; NHL, non-Hodgkin lymphoma; NOS, not otherwise specified; DLBCL, diffuse large B-cell lymphoma; FL, follicular lymphoma; BL, Burkitt’s lymphoma; CLL/SLL, chronic lymphocytic leukemia/small lymphocytic lymphoma; MALT, mucosal-associated lymphoid tissue; PTCL, peripheral T-cell lymphoma; ALCL, anaplastic large cell lymphoma; LPL, lymphoplasmacytic lymphoma.

### Survival Analysis

**Figure 1:**
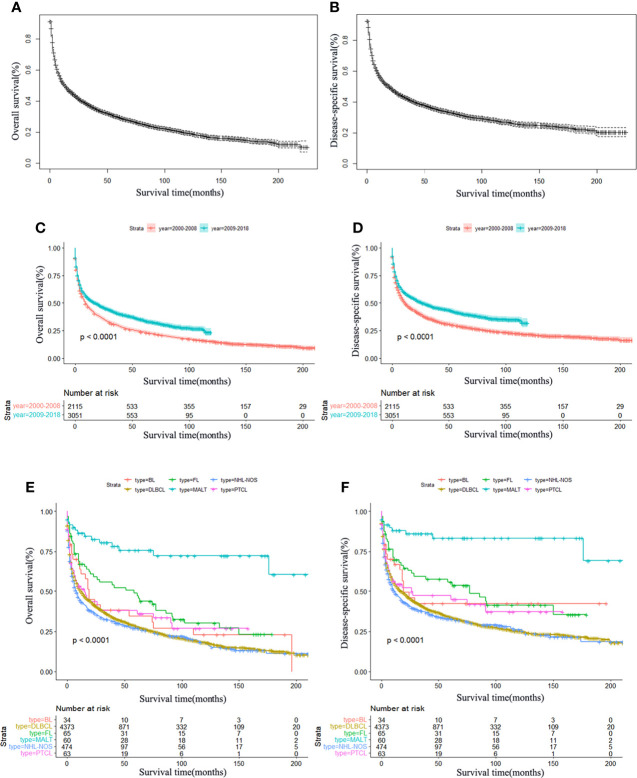
Survival analysis of primary central nervous system lymphoma. OS **(A)** and DSS **(B)** curves for all patients. Survival analysis according to the year of diagnosis; OS **(C)** and DSS **(D)** have significantly improved in the past decades, *P* < 0.0001. Survival curves of OS **(E)** and DSS **(F)** according to the main histological subtypes. MALT, mucosal-associated lymphoid tissue; DLBCL, diffuse large B-cell lymphoma; BL, Burkitt’s lymphoma; FL, follicular lymphoma; PTCL, peripheral T-cell lymphoma; NHL, non-Hodgkin lymphoma; NOS, not otherwise specified.

There were 3,660 patients who died during the follow-up period, and 3,101 deaths were disease-specific. Kaplan–Meier curves illustrating OS and DSS are shown in **Figures 1A, B**. The median OS and DSS were 13.0 and 19.0 months. The 1-, 3-, and 5-year OS were 50.1%, 36.0%, and 27.2%, respectively, and the corresponding DSS were 54.4%, 41.3%, and 33.5%, respectively. Patients diagnosed in 2009 to 2018 showed better OS and DSS than those patients diagnosed in 2000 to 2008 (*P* < 0.0001) (**Figures 1C, D**).

**Figure 2:**
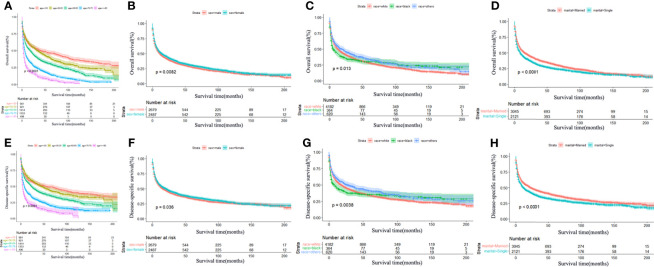
Survival analysis of primary central nervous system lymphoma stratified by age, sex, race, and marital status. Significant statistical difference was found in OS with age **(A)**, *P* < 0.0001; sex **(B)**, *P* = 0.0082; race **(C)**, *P* = 0.0013; and marital status **(D)**, *P* < 0.0001. Significant statistical difference was found in DSS with age **(E)**, *P* < 0.0001; sex **(F)**, *P* = 0.036; race **(G)**, *P* = 0.0038; and marital status **(H)**, *P* < 0.0001. Inferior OS and DSS were significantly associated with older age, male, black, and single status.

In the whole cohort, the best 3-year OS and DSS were observed in MALT (OS: 78.3%, DSS: 86.0%) and FL (OS: 53.8%, DSS: 57.4%). In addition, the Kaplan–Meier survival curves for OS and DSS of the main histological subtypes are presented in **Figures 1E, F**. Furthermore, Kaplan–Meier survival analysis was also performed stratifying patients according to sex, age, race, marital status, and treatment. It was shown that increasing age was significantly associated with worse OS and DSS (**Figures 2A, E**). Females had significantly better OS and DSS than males (**Figures 2B, F**). Patients who were others (American Indian/Alaskan Native or Asian/Pacific Islander) (**Figures 2C, G**) and married (**Figures 2D, H**) had better OS and DSS according to the univariate analysis.

**Figure 3:**
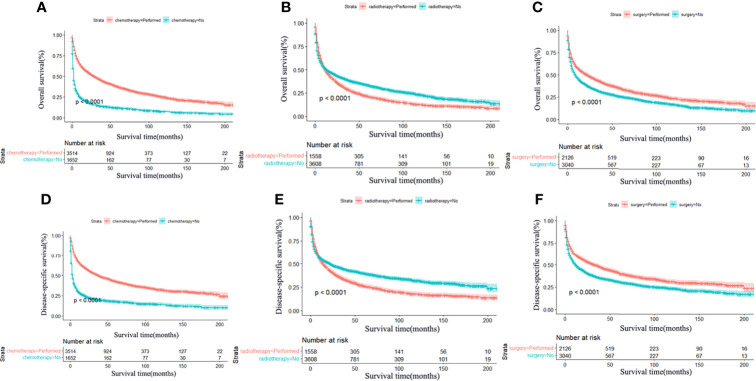
Survival analysis of primary central nervous system lymphoma stratified by treatment: chemotherapy, radiotherapy, and surgery. Significant statistical difference was found in OS and DSS between patients with chemotherapy and no chemotherapy **(A, D)**, radiotherapy and no radiotherapy **(B, E)**, and surgery and no surgery **(C, F)**, *P* < 0.0001. Patients who underwent chemotherapy and surgery achieved significantly longer OS and DSS compared with those who did not. However, radiotherapy led to worse OS and DSS in the long term.

**Figure 4:**
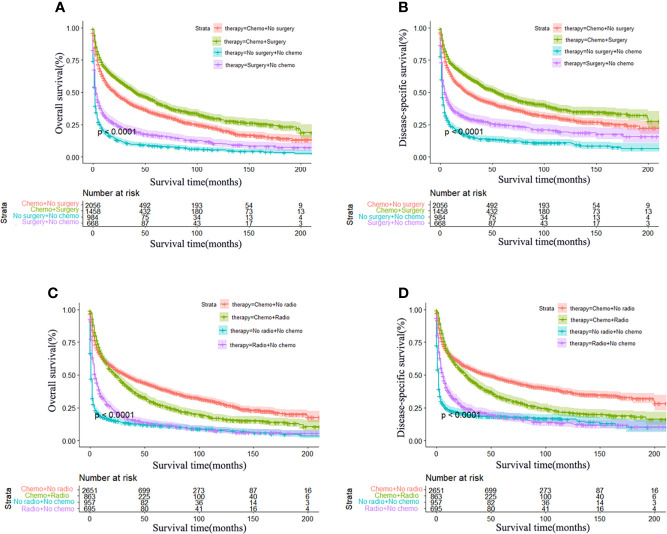
Effect of combination therapy on primary central nervous system lymphoma. Kaplan–Meier survival curves of the combined effect of chemotherapy and surgery: OS **(A)** and DSS **(B)** and of chemotherapy and radiotherapy: OS **(C)** and DSS **(D)**. Surgery combined with chemotherapy was significantly associated with better OS and DSS, *P* < 0.0001. Chemotherapy combined with radiotherapy was associated with better OS and DSS in the early stage of the treatment; however, the long-term OS and DSS were not superior to chemotherapy alone, *P* < 0.0001.

In terms of treatment, patients who underwent chemotherapy (**Figures 3A, D**) or surgical resection (**Figures 3C, F**) achieved significantly longer OS and DSS. However, radiotherapy led to worse OS and DSS in the long term (**Figures 3B, E**). In addition, a total of 2,126 patients underwent surgical resection, consisting of 600 total resection, 1,331 partial resection, and 195 unknown operative method. We further explored the partial resection and total resection on PCNSL outcome and found that total resection was associated with a survival benefit over partial resection (*P* < 0.0001) (**Supplementary Material**). We also explored the effects of combination therapy and found that surgical resection combined with chemotherapy was associated with better OS and DSS (**Figures 4A, B**), but radiotherapy combined with chemotherapy led to worse OS and DSS in the long term (**Figures 4C, D**). Finally, we performed multivariate Cox regression analysis and revealed that race, sex, age, marital status, surgical resection, and chemotherapy were independent predictors of OS and DSS, but radiotherapy was only an independent prognostic factor for OS (**Table 3**).

**Table 3:** Multivariable Cox regression analysis of the independent prognostic factors for OS and DSS among PCNSL patients.

Variables	Overall survival			Disease-specific survival		
	HR	95% CI	*P*	HR	95% CI	*P*
Race						
White	Reference			Reference		
Black	1.21	1.06–1.39	0.005	1.25	1.08–1.44	0.003
Others	0.92	0.83–1.03	0.138	0.91	0.81–1.02	0.094
Sex						
Male	Reference			Reference		
Female	0.84	0.78–0.89	<0.001	0.86	0.80–0.93	<0.001
Age						
<50	Reference			Reference		
50–59	1.48	1.31–1.67	<0.001	1.32	1.16–1.50	<0.001
60–69	1.90	1.70–2.13	<0.001	1.69	1.50–1.90	<0.001
70–79	2.65	2.37–2.96	<0.001	2.28	2.03–2.56	<0.001
≥80	3.32	2.91–3.79	<0.001	2.76	2.39–3.19	<0.001
Years of diagnosis						
2000–2008	Reference			Reference		
2009–2018	0.79	0.74–0.84	<0.001	0.77	0.72–0.83	<0.001
Marital status						
Married	Reference			Reference		
Single	1.21	1.13–1.30	<0.001	1.22	1.13–1.31	<0.001
Classification						
Aggressive B-cell NHL	Reference			Reference		
Indolent B-cell NHL	0.33	0.27–0.42	<0.001	0.31	0.24–0.40	<0.001
T-cell NHL	0.82	0.63–1.07	0.148	0.80	0.59–1.07	0.132
NHL-NOS	0.96	0.86–1.08	0.501	0.95	0.85–1.07	0.432
Others	1.08	0.52–2.28	0.834	1.09	0.49–2.43	0.838
Surgery						
Performed	Reference			Reference		
No/unknown	1.34	1.25–1.43	<0.001	1.36	1.26–1.46	<0.001
Chemotherapy						
Performed	Reference			Reference		
No/unknown	2.73	2.53–2.94	<0.001	2.59	2.39–2.81	<0.001
Radiation						
Performed	Reference			Reference		
No/unknown	1.12	1.04–1.20	0.003	1.08	1.00–1.17	0.054

### Construction of the Nomogram

**Figure 5:**
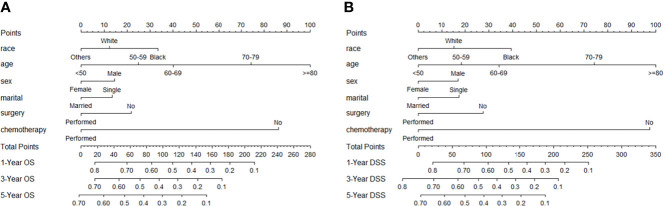
Nomogram to predict 1-, 3-, and 5-year OS **(A)** and DSS **(B)** probability in patients with primary central nervous system lymphoma. The OS and DSS rates at 1, 3, and 5 years can be predicted by integrating scores related to race, age, sex, marital status, surgery, and chemotherapy and projecting the total points to the bottom scale.

**Figure 6:**
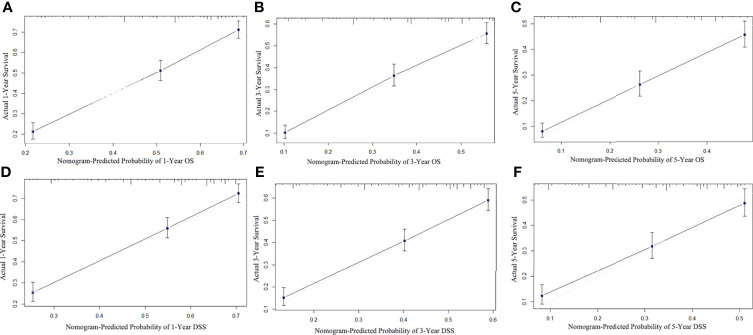
Calibration curve of the nomogram for the prediction of 1-, 3-, and 5-year OS **(A–C)** and DSS **(D–F)**. The abscissa represents the nomogram-predicted survival rate, the ordinate represents the actual survival rate, and the calibration curves for 1-, 3-, and 5-year survival rates showed satisfactory agreements between the predicted and actual values.

Considering that DLBCL was the main histological subtype of PCNSL, we developed a prediction model specifically for DLBCL patients. DLBCL (*n* = 4,373) patients were randomly divided into the training cohort (*n* = 3,061) and the validation cohort (*n* = 1,312) in a ratio of 7:3 for model construction and validation. Firstly, univariate and multivariate Cox regression analyses were conducted to select the independent prognostic factors for OS and DSS. The univariate and multivariate analysis results are displayed in **Table 4**. These significant prognostic factors from univariate Cox regression analysis were incorporated into multivariate analysis. Significant predictors of OS and DSS on multivariate analysis were used to establish the nomograms. The OS and DSS nomograms at 1, 3, and 5 years are shown in **Figure 5**. Then, the nomogram performance was assessed with discrimination and calibration by using the external validation cohort. The C-index values of OS and DSS were 0.704 (95% CI 0.687–0.721) and 0.698 (95% CI 0.679–0.717), indicating the high discrimination ability of the nomograms. The calibration curves of the training cohort and the external validation cohort are presented in **Figure 6**, which represented good agreement among the predicted survival and the actual survival at 1, 3, and 5 years.

**Table 4:** Univariate and multivariate Cox regression analyses of the ability of each factor in predicting OS and DSS among DLBCL patients.

	Overall survival			Disease-specific survival		
	HR	95% CI	*P*	HR	95% CI	*P*
Univariate analyses						
Race						
White vs. black	1.16	1.00–1.34	0.046	1.21	1.04–1.42	0.014
White vs. others	0.85	0.76–0.95	0.004	0.83	0.73–0.94	0.003
Sex						
Male vs. female	0.92	0.86–0.99	0.021	0.93	0.86–1.00	0.054
Age						
0–50 vs. 50–59	1.05	0.92–1.20	0.453	0.94	0.82–1.07	0.342
0–50 vs. 60–69	1.39	1.23–1.55	<0.001	1.22	1.08–1.38	0.001
0–50 vs. 70–79	2.19	1.96–2.45	<0.001	1.89	1.68–2.13	<0.001
0–50 vs. ≥80	3.25	2.83–3.73	<0.001	2.73	2.35–3.16	<0.001
Marital status						
Married vs. single	1.23	1.14–1.32	<0.001	1.27	1.18–1.37	<0.001
Surgery						
Performed vs. no/unknown	1.27	1.18–1.37	<0.001	1.30	1.21–1.41	<0.001
Chemotherapy						
Performed vs. no/unknown	3.17	2.95–3.41	<0.001	3.03	2.80–3.28	<0.001
Radiation						
Performed vs. no/unknown	0.83	0.77–0.89	<0.001	0.80	0.74–0.87	<0.001
Multivariate analyses						
Race						
White vs. black	1.17	1.01–1.36	0.042	1.18	1.00–1.39	0.048
White vs. others	0.88	0.79–0.99	0.028	0.86	0.76–0.97	0.017
Sex						
Male vs. female	0.84	0.78–0.90	<0.001	0.86	0.79–0.93	<0.001
Age						
0–50 vs. 50–59	1.35	1.18–1.54	<0.001	1.20	1.04–1.38	0.013
0–50 vs. 60–69	1.74	1.54–1.96	<0.001	1.53	1.35–1.74	<0.001
0–50 vs. 70–79	2.43	2.16–2.74	<0.001	2.10	1.85–2.38	<0.001
0–50 vs. ≥80	3.03	2.63–3.50	<0.001	2.55	2.18–2.97	<0.001
Marital status						
Married vs. single	1.21	1.12–1.31	<0.001	1.23	1.13–1.33	<0.001
Surgery						
Performed vs. no/unknown	1.32	1.22–1.42	<0.001	1.34	1.24–1.45	<0.001
Chemotherapy						
Performed vs. no/unknown	2.97	2.74–3.22	<0.001	2.80	2.56–3.05	<0.001
Radiation						
Performed vs. no/unknown	1.08	1.00–1.17	0.053	1.04	0.95–1.13	0.394

### Web-Based Survival Rate Calculator

**Figure 7:**
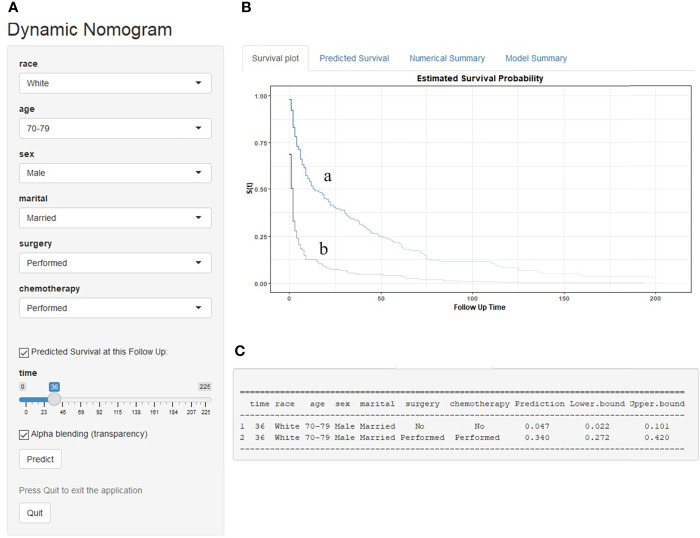
An example to illustrate the use of the web-based survival rate calculator. **(A)** A 75-year-old married white man was diagnosed as PCNSL with DLBCL; if he refused chemotherapy and surgery, his 3-year OS rate is approximately only 4.7% (95% CI 0.022–0.101) (C); if he was given chemotherapy and surgery, his 3-year OS rate is approximately 34.0% (95% CI 0.272–0.420) **(C)**. **(B)** His survival curve depending on whether he was treated or not: received treatment (a) and refused treatment (b).

A dynamic web-based survival rate calculator based on the nomogram was established to predict the long-term OS (https://tangdongshengarticle.shinyapps.io/DynNomapp/). For instance, a 75-year-old white married man was diagnosed as PCNSL with DLBCL; if he refused surgical resection and chemotherapy, his 3-year OS rate is approximately only 4.6% (95% CI 0.022–0.101); if he was given surgical resection and chemotherapy, his 3-year OS rate is approximately 34.0% (95% CI 0.272–0.420) (**Figure 7**).

## Discussion

PCNSL represents a rare but highly aggressive NHL with poor prognosis. In light of the low incidence of PCNSL, the current understanding of PCNSL is mainly based on retrospective analysis with small series. Therefore, we conducted a study based on a large population (SEER database) and 5,166 PCNSL patients were included.

The mean age at diagnosis was 63.1 ± 14.9 years, and the male to female ratio was 1.1:1.0, which was largely consistent with a population-based study from Australia (14). Previous studies have demonstrated that age was a significant and adverse prognostic factor (15–17). In the present study, inferior OS and DSS were significantly associated with older age, consistent with previous results. Interestingly, our study revealed that married patients tend to have better outcomes than single patients (including divorced/widowed/separated patients), which was consistent with another population-based study (18). The mechanism between marital status and survival is unclear, social–psychological factors may contribute to it (19), and married patients may have better socioeconomic status and more emotional support than single patients.

Although the introduction of HD-MTX has significantly improved PCNSL prognosis, numerous patients still died of treatment-related mortality, chemotherapy resistance, and disease relapse (20). The recent progress in understanding the pathophysiology of PCNSL has led to novel therapeutics introduced into clinical trials that have shown promising results (21). Based on population analysis, we found that patients diagnosed between 2009 and 2018 had better OS and DSS than those diagnosed 2000–2008, which reflected the development of novel therapies.

PCNSL is characterized by a frequent early wide dissemination and the involvement of a deep part of the brain that leads to poor efficacy of surgery (22). Previous research suggested that surgical resection (including complete and partial surgical resection) has no significant survival advantage and was even associated with higher mortality that should be avoided (23–26). The role of surgery is only to establish a diagnosis by stereotactic biopsy. However, with a large number of applications of new techniques and practices in recent years, including increased use of MRI, frameless stereotyping, and tumor visualization, effectiveness and tolerability have greatly improved. Survival advantage was proven and the traditional view has been questioned in some studies (27–29). This study showed that surgical resection might play a role in significantly improving OS and DSS and was an independent risk factor for survival. Moreover, combining surgical excision and chemotherapy can bring favorable OS and DSS than chemotherapy alone, which suggested that multimodal therapy may be more beneficial. At present, there is insufficient evidence to recommend surgical resection for PCNSL, and several small population retrospective studies indicated that specific subgroups of patients with single lesions and superficial tumors might gain a survival benefit from resection (30, 31). Our study also showed the advantages of surgical resection based on a large population. However, the role of surgical resection should be re-evaluated in large-scale prospective research.

Due to high sensitivity to radiation, newly diagnosed PCNSL patients have historically received whole-brain radiotherapy (WBRT). However, WBRT‐associated delayed neurotoxicity has limited its use, especially for age older than 60 years (7, 32, 33). Given the higher risk of neurotoxicity and the limited durability of treatment responses, WBRT is not considered as the standard initial therapy for PCNSL patients (34, 35). Recently, many clinical studies have engaged in whether different radiotherapy regimens (including reduced dose and partial‐brain radiotherapy) in combination with chemotherapy can bring better clinical outcomes (36–40). However, the results remain controversial. Based on a large population analysis, radiotherapy did not improve long-term effects and was associated with inferior OS and DSS compared with no radiotherapy according to the univariate analysis. Multivariate analysis revealed that radiotherapy was an independent prognostic factor for OS, but not for DSS. We further explored the combination of radiotherapy and chemotherapy and found that patients may benefit from combination therapy in the early stage of the treatment; unfortunately, the long-term outcomes were not superior to chemotherapy alone because of the high incidence of delayed neurotoxicity. The benefit of radiotherapy in establishing local control of tumors must be weighed against the increased risk of long‐term neurotoxicity. In our opinion, radiotherapy may not be the preferred consolidation therapy strategy, autologous stem-cell transplantation (ASCT) may be the better choice for appropriate patients (41), and radiotherapy has a role in the treatment of PCNSL patients who cannot tolerate chemotherapy or ASCT. Due to unknown information about detailed radiotherapy and chemotherapy regimens, subgroup analysis could not be performed. Therefore, these results should be interpreted cautiously.

The nomogram has become a useful tool for clinical decision-making and visualization and quick risk assessment for clinicians. In this study, it was found that race, age, sex, marital status, chemotherapy, and surgical resection were independent prognostic factors for OS and DSS in DLBCL patients, and we constructed the nomograms to predict 1-, 3-, and 5-year survival based on these factors. The significantly higher C-index 0.704 and 0.698 of the nomograms proved discriminative power. Moreover, the calibration curve exhibited good consistency among the predicted survival and the actual survival. However, due to manual calculations, the nomograms are not easy to apply in clinical practice, so we further developed a dynamic web-based survival rate calculator that can predict the long-term OS dynamically at different time points for DLBCL patients based on the nomogram (https://tangdongshengarticle.shinyapps.io/DynNomapp/).

This study has several limitations. Firstly, potential biases were unavoidable as a retrospective study. Secondly, other potential prognostic factors, such as Karnofsky performance status score, size and number of lesions, and LDH level, were not registered in the SEER database and these factors cannot be combined to predict prognosis. Thirdly, detailed chemotherapy and radiotherapy regimens for patients were not available. We were unable to further analyze the impact of different treatment regimen on prognosis. Lastly, the nomograms were established and verified by using the same database, so it was necessary to prospectively verify the nomogram in another independent data set for reliable evaluation. Therefore, the results of the present study should be interpreted with caution due to the above limitations. However, our study still provided useful information and important insights on PCNSL despite these limitations based on a large population.

In conclusion, age, race, sex, marital status, use of chemotherapy, and surgical resection were independent prognostic factors for OS and DSS, but radiotherapy was only for OS based on the SEER database. Surgical resection might have a therapeutic benefit for PCNSL patients. Radiotherapy was effective in the therapeutic initial stage, but the long-term outcome was not satisfactory. We also developed a predictive nomogram and a web-based survival rate calculator to predict the long-term OS in DLBCL patients, which showed favorable applicability and accuracy that could help in the prediction of mortality and the treatment choice.

## Data Availability Statement

Publicly available datasets were analyzed in this study. These data can be found here: https://seer.cancer.gov.

## Author Contributions

LY and CW designed the study. DT and YC wrote the manuscript. YS and HT prepared the images. All the authors contributed to the data collection and data analysis, critically revised the manuscript, and approved the final version of the paper.

## Funding

This study was funded by Jiangsu Commission of Health (H2019082).

## Conflict of Interest

The authors declare that the research was conducted in the absence of any commercial or financial relationships that could be construed as a potential conflict of interest.

## Publisher’s Note

All claims expressed in this article are solely those of the authors and do not necessarily represent those of their affiliated organizations, or those of the publisher, the editors and the reviewers. Any product that may be evaluated in this article, or claim that may be made by its manufacturer, is not guaranteed or endorsed by the publisher.

## Supplementary Material

Supplementary Figure 1 **(A)** The effect of extent of surgery on OS in PCNSL patients. **(B)** The effect of extent of surgery on DSS in PCNSL patients. The total resection was significantly associated with better OS and DSS than partial resection, *P* < 0.0001.
