# Supplementary figures and images for: Epidemiologic Characteristics, Prognostic Factors, and Treatment Outcomes in Primary Central Nervous System Lymphoma: A SEER-Based Study
Source: Front Oncol. 2022 Feb 10;12:817043. doi: 10.3389/fonc.2022.817043 (PMC8867087; doi:10.3389/fonc.2022.817043)

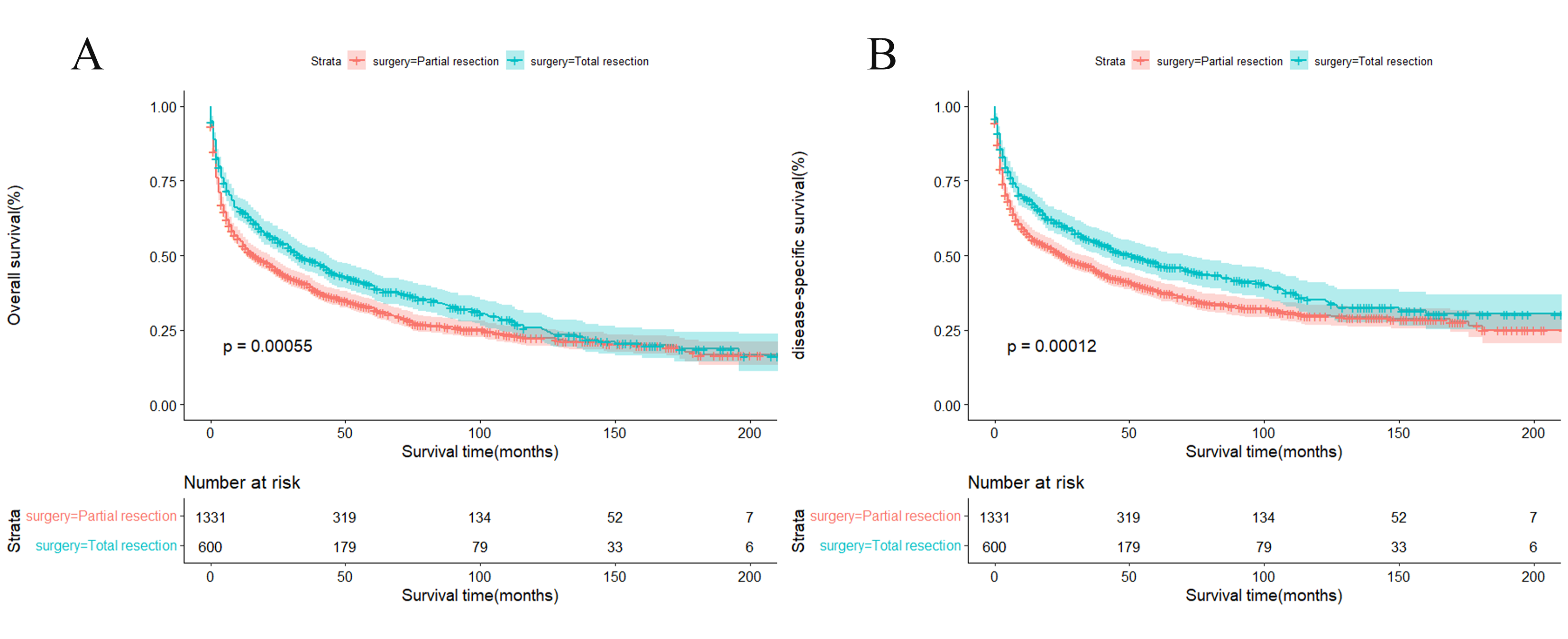

Supplement: Supplementary Figure 1 — (A) The effect of extent of surgery on OS in PCNSL patients. (B) The effect of extent of surgery on DSS in PCNSL patients. The total resection was significantly associated with better OS and DSS than partial resection, P < 0.0001. [file Image_1.tif]
